# Desensitization in HLA-incompatible kidney transplant recipients: current strategies and emerging perspectives

**DOI:** 10.1093/ckj/sfaf219

**Published:** 2025-07-09

**Authors:** Mahmut Altindal, Mustafa Guldan, Lasin Ozbek, Sama Mahmoud Abdel-Rahman, Selen Unlu, Ahmet Murt, Nuri B Hasbal, Abdulmecit Yildiz, Charles J Ferro, Adrian Covic, Caner Süsal, Mehmet Kanbay

**Affiliations:** Department of Medicine, Section of Nephrology, Koc University School of Medicine, Istanbul, Turkey; Department of Medicine, Koc University School of Medicine, Istanbul, Turkey; Department of Medicine, Koc University School of Medicine, Istanbul, Turkey; Department of Medicine, Koc University School of Medicine, Istanbul, Turkey; Department of Medicine, Koc University School of Medicine, Istanbul, Turkey; Division of Nephrology, Department of Internal Medicine, Cerrahpasa Medical Faculty, Istanbul University—Cerrahpasa, Istanbul, Turkey; Department of Medicine, Section of Nephrology, Koc University School of Medicine, Istanbul, Turkey; Division of Nephrology, Faculty of Medicine, Bursa Uludag University, Nilufer, Bursa, Turkey; Department of Renal Medicine, University Hospitals Birmingham and Institute of Cardiovascular Sciences, University of Birmingham, Birmingham, UK; Nephrology Clinic, Dialysis and Renal Transplant Center, “C.I. Parhon” University Hospital, Iasi, Romania; Transplant Immunology Research Center of Excellence TIREX, Koç University, Istanbul, Turkey; Department of Medicine, Section of Nephrology, Koc University School of Medicine, Istanbul, Turkey

**Keywords:** CAR T-cell therapies, desensitization, kidney transplantation, plasma cell-directed therapies, plasmapheresis

## Abstract

Despite development of kidney paired donation programs and prioritization in kidney allocation schemes, transplantation rates are still low and waiting times remain prolonged for highly sensitized kidney transplant recipients with broad human leukocyte antigen antibody reactivity. Desensitization confers an invaluable option improving access to kidney transplantation for sensitized patients who could not benefit from kidney paired donation programs and kidney allocation schemes. Conventional desensitization strategies use intravenous immunoglobulin combined with either plasmapheresis or monoclonal anti-CD20 antibodies. Imlifidase, IL-6 targeting agents, plasma cell-directed therapies, complement inhibitors, chimeric antigen receptor T-cell therapies, and B cell-activating factor inhibitors are emerging new options in the hope of enhancing and sustaining the efficacy of desensitization to improve allograft longevity. In this review, we discuss the rationale and outcome of desensitization with various strategies alone or in combination. Our aim is also to provide some insight for decision when pursuing desensitization might be successful or futile in sensitized patients.

## INTRODUCTION

Transplant candidates exposed to non-self-antigens via transfusion, pregnancy, previous transplants, or infections may develop alloantibodies to human leukocyte antigens (HLA), a process termed sensitization [1]. Despite ongoing rigorous efforts to prioritize highly sensitized (HS) candidates in the transplant allocation systems, transplantation rates remain low and waiting times prolonged for these patients due to frequently positive crossmatch results [2, 3].

**Figure 1:**
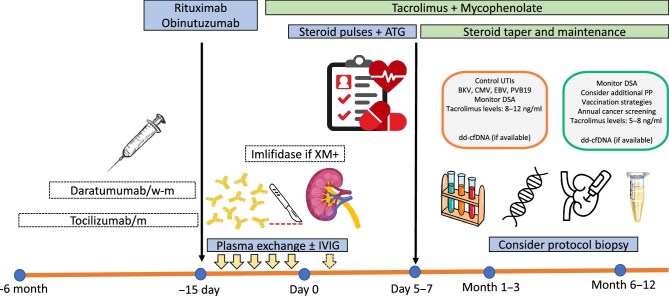
Desensitization strategies and post-transplant monitoring in patients undergoing HLA-incompatible KT. w, weekly; m, monthly; UTI, urinary tract infections; BKV, BK virus; CMV, cytomegalovirus; EBV, Ebstein–Barr virus; PVB19, parvovirus B19; dd-cfDNA, donor-derived cell-free DNA.

**Figure 2:**
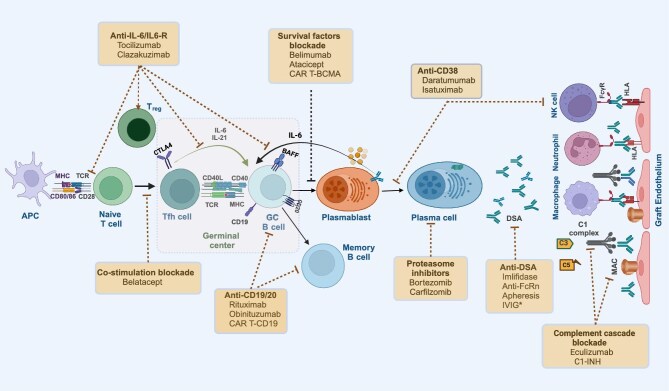
Crucial steps toward institution of B-cell alloimmunity and action sites of multiple desensitization strategies interfering immune effector mechanisms. APCs present HLA antigens to naive T cells. Activated T and B cells migrate to GC and the former differentiate into Tfh cells. These cells stimulate proliferation and differentiation of GC B cells, which lead to generation of memory B cells or antibody-producing short- (plasmablasts) and long-lived plasma cells. Plasmablasts secrete IL-6, augmenting formation of more germinal centers. Binding of antibodies to the allograft endothelium may invoke NK cell, macrophage and neutrophil activation through FcγR and induce complement activation via the classical pathway. Co-stimulation blockers, agents blocking survival factors and monoclonal antibodies acting through interleukin-6/IL-6 receptors may disrupt GC formation, hinder B-cell activation and differentiation, hamper the formation or durability of plasma cells. CD38 monoclonal antibodies interfere with antibody-producing plasma cells and NK cells while proteosome inhibitors deplete plasma cells. Complement inhibitors either disrupt the C1 complex or block the terminal component C5 and the formation of the MAC. DSAs can be addressed directly either by cleaving IgG with imlifidase or antagonizing the FcRn that accelerates the catabolism of IgG. *The immunomodulatory activity of IVIG is provided by multiple inhibitory actions on B and T cells, and complement cascade activation, antibody formation, and recycling. APC, antigen presenting cell; CP, classical pathway; FcγR, Fc gamma receptor; FcRn, Fc neonatal receptor; LLPC, long-lived plasma cells; MAC, membrane attack complex.

Currently, the available options for HS patients include kidney paired donation (KPD) programs, waiting for a compatible deceased-donor (DD) offer or proceed with an HLA antibody incompatible (HLAi) living donor kidney transplant with pre-transplant desensitization. Up to now, various desensitization protocols have been employed, and the method chosen is commonly guided by immunologic phenotyping assessing the specificity and strength of the patient's donor-specific HLA antibodies (DSA). Currently applied desensitization strategies and their cellular and molecular targets are depicted in Figs 1 and 2, respectively. In this review, our aim is to display the philosophy and outcome of desensitization protocols. We offer some insight into when attempting desensitization might be successful or futile. Novel desensitization strategies will also be reviewed.

## HLA SENSITIZATION

### Estimating the degree of sensitization

The methods used to evaluate the degree of sensitization include determination of broadness of HLA antibody reactivity and the strength of DSA by antibody testing and crossmatching. Complement-dependent cytotoxicity crossmatch (CDC-XM) and the more sensitive flow cytometry crossmatch (FC-XM) tests estimate the relative amount of DSA in the potential recipient's serum. Historically cell-based panel reactive antibody (PRA) testing was performed to evaluate the breadth of sensitization. The introduction of solid-phase assays (SPA) revolutionized the detection and characterization of HLA antibodies which promoted the establishment of novel tools to interpretate and estimate the degree of sensitization such as virtual or calculated PRA (vPRA, cPRA) and calculated reaction frequency (cRF) [4–8]. These tools, which replaced older cell-based PRA testing, have become the essential components in organ allocation schemes, KPD programs, and immunological risk stratification algorithms. They are dependent on which HLA antigens will be considered unacceptable, and are center-specific, based on risk tolerance for preformed DSA detected by SPA. There is no universally accepted threshold for definition of sensitization; nevertheless, many transplant centers consider candidates with a cPRA ≥ 30% as sensitized, whereas a cPRA ≥ 80% and a cPRA ≥ 98% are widely accepted as HS and very HS, respectively [9, 10].

### Clinical implications of HLA antibody testing

A positive CDC T-cell XM, when an autoantibody has been excluded, has traditionally been accepted as a contraindication to kidney transplantation (KT) without desensitization due to very poor transplant outcomes [11]. Attempts to relate mean fluorescence intensity (MFI) values acquired from solid phase single antigen bead (SAB) assays with CDC-XM and FC-XM tests revealed that the highest MFI value usually predicted CDC-XM positivity [12–14]. There has been substantial discussion and debate about the clinical implications of low titer HLA antibodies that are not associated with a positive CDC-XM and FC-XM [15, 16]. C1q binding may vary across antibody subgroups and C1q-positive DSA may imply a potential risk for adverse graft outcomes, however, complement activation is largely a consequence of a high concentration of DSA [17–20].

### Assessment of desensitization efficacy

The efficacy of desensitization may be estimated by various means, including the rate of transplant access, conversion of a crossmatch test from positive to negative, titration studies, reduction in MFI levels, decrease in number of unacceptable antigens, and cPRA [13]. Advantages and disadvantages of these parameters are summarized in Table 1.

**Table 1:** Advantages and disadvantages of parameters for measuring the efficacy of desensitization.

	Pros	Cons
Mean florescence intensity	§ Readily available at many centers and relatively easy to estimate	§ Bead saturation, serum interference, difficult to interpret among patients with multiple antibodies
Number of unacceptable antigens	§ Easy to estimate	§ Similar limitations as MFI § May underestimate (HS patients) or overestimate the effect of treatments
Crossmatches	§ Clinically relevant outcome among candidates for LDKT	§ Requires the donor
Transplantation	§ Clinically relevant outcome	§ Affected by other factors than antibody reduction such as comorbidities, donor availability
Neat cPRA	§ Simplifies complex data § Related to probability of receiving an organ	§ Not sensitive to changes in antibodies among HS patients § Could be unreliable if based on neat MFI. § An alteration in a common antibody may inordinately alter cPRA
cPRA combined with titers	§ Sensitive to changes in antibodies among the ones likely benefit from desensitization. § Functional to incorporate complex antibody data	§ Costly and labor intensive

*Adapted from Tambur *et al.* [13].

### Outcome after desensitization

Contrary to previous reports from the USA, a UK study by Manook *et al.* found that desensitization does not confer a survival advantage compared to remaining on the waiting list for a compatible organ [21–23]. Trials investigating the survival benefit of desensitization over staying on the waiting list are summarized in Table 2 [21–24]. The obtained data altogether suggest that the survival benefit of HLAi-living donor kidney transplantation (HLAi-LDKT) is not universal [25, 26]. It could vary individually depending on medical comorbidities, average waiting times for deceased organ offer, efficacy and size of KPD programs, local dialysis mortality rates, and the selection criteria for desensitization [24, 27, 28]. In a multicenter US study, Orandi *et al.* found that major determinant of the survival benefit of HLAi-LDKT was the antibody strength, which was stratified as pre-transplant positive SAB and negative FC-XM, positive FC-XM and negative CDC-XM, positive CDC-XM [29]. Moreover, possible complications of HLAi-kidney transplantation (HLAi-KT) with desensitization, which may involve severe episodes of rejection, infections, and costly hospitalizations, may have significant psychological and even psychiatric implications [28].

**Table 2:** Recent studies investigating the survival benefit of desensitization over staying on the waiting list.

Study	Study population	Numbers	5-year survival (%)	Survival benefit
Koo *et al.* [24]	Adopted cases from two participating centers in Korea and controls from the entire Korean waiting list population	189 desensitized	98.3	
		1 860 on waiting list:		Yes
		50% transplanted	90.5	
		50% dialysis only	89.6	
Manook *et al.* [21]	Both cases and controls adopted from the entire UK waiting list population and included controls who subsequently received a living donor transplant	213 desensitized	92	
		852 on waiting list:		No
		59% transplanted	92	
		41% dialysis only	90	
Orandi *et al.* [22]	Cases from 22 participating centers in the USA and controls from the entire US waiting list population were included and controls subsequently received a living donor transplant were excluded	1 025 desensitized	86	
		10 250 on waiting list:		Yes
		50% transplanted	74	
		50%: dialysis only	50	
Montgomery *et al*.^a^ [23]	Included cases from a single center in the USA and controls from the entire US waiting list population	211 desensitized	81	
		2 090 on waiting list:		
		50% transplanted	66	Yes
		50%: dialysis only	52	

^a^Adapted from Süsal *et al.* and Clayton *et al.* [25, 26].

### Patient groups who may benefit from desensitization

Given the conflicting data regarding survival benefit and the need for case-by-case evaluation; the decision to proceed with desensitization mainly rests on the pre-transplant HLA antibody strength, transplant center's expertise, available facilities and resources, predicted waiting times for a DD organ based on the magnitude of local donor pool, and the allocation system. Notwithstanding, patient groups that are most likely to benefit from desensitization are HS patients with cPRA > 99%, HS patients with cPRA > 98% who have been either on the waiting list >5 years, and those who could have not achieved a compatible offer through a PKD program for a considerable time after several match runs [25, 30]. Presensitized transplant candidates living in countries with very limited donor pools or higher dialysis mortality rates as well as patients with rare HLA types, especially if not HS, also benefit strongly from desensitization [25, 30].

## CONVENTIONAL METHODS FOR DESENSITIZATION

### Antibody removal

Therapeutic apheresis has been used to enable successful transplantation in crossmatch-positive patients; and for many units, it is still the mainstay of desensitization prior to KT [31–33]. Noble *et al.* evaluated the efficacy and tolerance for different therapeutic apheresis techniques including plasmapheresis (PP), double filtration plasmapheresis (DFPP), and immunoadsorption in HS candidates for KT [34]. A total of 881 sessions were performed in 45 patients. The apheresis procedures enabled HLAi-KT in 39 patients. Immunoadsorption, PP, and a lower maximal pre-transplant DSA MFI were associated with a greater decrease in class II DSA. Overall, apheresis was well tolerated, and its safety was acceptable; however, DFPP was associated more frequently with hypotension and severe adverse events (6.5%) than immunoadsorption and PP (1.2% and 1.9%, respectively). Malvezzi *et al.* suggested the use of PP for cases undergoing desensitization with an MFI of the highest DSA between >3000 and ≤9000, the use of DFPP for cases with an MFI between >9000 and ≤12 000, and the use of immunoadsorption for cases with an MFI of >12 000 [35]. For desensitization prior to HLAi-LDKT, some transplant centers prefer a protocol that consists of low-dose ІVΙG (100 mg/kg after each PP session) in combination with alternate-day PP [23, 31, 36, 37]. There are no prospective randomized trials comparing the use of PP/low-dose intravenous immunoglobulin (IVIG) protocol with high-dose IVIG (2 g/kg IVIG without PP) for desensitization. However, one retrospective study compared three different desensitization regimens in KT recipients with a positive T-cell CDC-XM. The authors concluded that PP/low-dose IVIG is more likely to achieve desensitization in patients with crossmatch titers in the 1:8 to 1:16 range when compared to a single high-dose IVIG without PP. Humoral rejection rates among patients transplanted were 80%, 37%, and 29% for patients receiving high-dose IVIG, PP/low-dose IVIG/rituximab, and PP/low-dose IVIG/rituximab/rATG-thymoglobulin, respectively. The authors also demonstrated that none of these three desensitization protocols were effective when the T-cell CDC-XM titers were >1:32 [38]. This study and others suggested that the primary determinant of desensitization outcomes and post-transplant antibody-mediated rejection (AMR) rates was DSA antibody strength, which also should be taken into account when comparing efficacy of different desensitization protocols [39, 40].

### Assessment of feasibility of antibody removal

The initial DSA strength can be used to anticipate effective desensitization. Although detection of pre-transplant DSA was shown to be associated with AMR and worse allograft outcomes [41], current SPAs have shortcomings with vital implications. Besides assay-specific parameters (i.e. denatured antigens), serum specific parameters including inhibitory factors, bead saturation, and the shared epitope phenomenon are the current hurdles that may affect reliability and accurate interpretation of the SPAs [42–44]. Given these flaws of SPAs, it has been demonstrated that antibody titers rather than MFI in undiluted untreated serum better reflect the antibody strength [17, 45, 46]. Several studies have reported that class II HLA antibodies, explicitly those directed against HLA-DQ, are more resistant to antibody removal treatment with PP/IVIG and proteasome inhibitors [37, 47–52]. In contrast, Pinelli *et al.* reported that, apart from HLA-A antibodies, antibodies to class I and class II HLA antigens respond equally well to pre-transplant desensitization with PP/IVIG [53]. However, reductions in titers decelerated with 4–5 PP/IVIG cycles, which indicates limited efficacy. The authors proposed that while HLA-DQ antibodies are reduced at a similar rate to antibodies against other loci, reduction in strength may be missed by MFI if both pre- and post-treatment antibodies saturate the beads in undiluted serum. In the same study, Pinelli *et al.* also showed that the feasibility of desensitization for LDKT could be guided by antibody titrations [53]. Titration studies also may serve to calculate how many treatment cycles are needed to lower DSA levels to a pre-transplant acceptable level and assessing the limits of desensitization to decide when attempts for desensitization are futile.

IVIG has been used as part of desensitization regimens in transplantation for decades. Most protocols use either high dose (2 g/kg) IVIG alone or low-dose IVIG (100 mg/kg) in combination with PP. The exact mechanism of its action is unknown. Several potential mechanisms have been proposed for its immunomodulatory activity including inhibition of complement activation, neutralization of anti-HLA alloantibodies, induction of anti-inflammatory cytokines and B-cell apoptosis, decreased pathogenic IgG survival (via interaction with FcRn or neonatal receptor), and interaction with macrophages and monocytes to induce the inhibitory FcgRIIb that act to mute antibody response [54, 55]. IVIG is also useful in preventing opportunistic infections in desensitized patients [56]. The only prospective RCT (NIH IG02 Trial) to assess the efficacy of IVIG-only strategy on allo-sensitization was performed 20 years ago and included 50 patients with PRA > 50%. IVIG 2 g/kg given monthly for 4 months or an equivalent volume of placebo with additional infusions at 12 and 24 months after entry if not transplanted and were followed for 30 months. The authors concluded that IVIG is better than placebo in reducing HLA antibody levels and improving transplantation rates in sensitized patients with kidney failure [57]. However, IVIG use alone for desensitization was insufficient to sustain low levels of anti-HLA antibody and is associated with antibody rebound post-transplantation with AMR [58, 59].

## B CELL-DIRECTED THERAPIES

DSAs derive from activation and ensuing maturation of naive B cells into alloantigen-specific memory B cells (mBCs) and antibody secreting cells embracing both short-lived blasts and long-lived plasma cells [60, 61]. Furthermore, B cells can process and present alloantigens to T cells, organize formation of tertiary lymphoid organs and modulate T-cell responses via cytokine secretion. In sensitized patients, to achieve a successful graft outcome, the therapeutic objective is not only to impede naive alloreactive B-cell activation but also to inhibit and eliminate mBCs and plasma cells.

### Anti-CD20 therapies

Rituximab as a chimeric monoclonal antibody (mAb) that binds to the CD20 molecule on immature and mature B lymphocytes [62] has been incorporated into the desensitization regimens with the goal of B-cell depletion in the context of KT [30, 39, 58, 59, 63]. A French prospective multicenter randomized controlled trial (RITUX-ERAH) in 38 patients failed to show benefit for rituximab (RTX) when added to standard of care (SOC) at 12 months in active AMR and it was associated with an increased incidence of opportunistic infections [64]. Indeed, in an extension of the same study, no benefit of RTX in addition with PP, IVIG, and steroids compared to placebo was observed 7 years after AMR [65]. Jackson *et al.* evaluated 256 post-transplant HLA antibody levels in 25 recipients desensitized with and 25 without RTX induction, to investigate the effect of B-cell depletion [66]. Even though RTX induction profoundly lowered the incidence and magnitude of HLA antibody rebound, it did not affect DSA elimination, AMR, or 5-year allograft survival when compared to recipients desensitized and transplanted without RTX [66]. Despite enabling peripheral blood B-cell depletion, RTX incompletely depletes lymphoid organ B cells.

Obinutuzumab, on the other hand, is a glycoengineered type II anti-CD20 mAb that has been demonstrated to be more efficacious than rituximab in peripheral blood and tissue B-cell depletion [67]. Obinituzumab use for desensitization was assessed in a phase I, open-label study (THEORY) including 25 HS kidney transplant candidates [68]. Although obinutuzumab plus IVIG resulted in peripheral B-cell depletion and reduced B cells in retroperitoneal lymph nodes; the decline in HLA antibodies, number of unacceptable antigens, and the cPRA scores were minimal and not clinically meaningful for most patients. Since depletion of CD20^+^ B cells alone did not have a significant influence on HLA antibody production, the authors suggested that HLA antibodies are produced by mature, long-lived plasma cells.

Current strategies deplete B cells unselectively regardless of their function and specificity. However, B cells, in form of regulatory B cells, also exhibit regulatory functions and inhibit alloimmune responses [69]. Thus, anti-CD 20 mAb use may impede some inhibitory pathways (i.e. regulatory B cells) in sensitized kidney allograft recipients that may lead to undesirable side-effects including a notable increase in acute T cell-mediated rejection within the first 3 months post-transplantation [60, 70]. Therapeutic expansion of regulatory B cells (B_reg_s) and possibly development of a B_reg_ cell therapy in the future would overcome this challenge [71].

## PLASMA CELL-DIRECTED THERAPIES

Late AMR (occurring 3 to 6 months after transplantation) shows distinct immunological characteristics and is more resistant to anti-rejection therapies than early AMR, provided by niche-resident plasma cells [51]. These bone marrow cells impart sustained memory for decades in humans [72]. Because long-lived plasma cells are the key producers of HLA antibodies, plasma cell-directed therapies were developed to target actions of these cells [73].

### Proteosome inhibitors

Bortezomib is a first generation, reversible protosome inhibitor [74] used to treat multiple myeloma. The favorable outcomes reported by early small, non-randomized studies that are confounded by use of conventional desensitization methods in conjunction with bortezomib were not supported by subsequent trials that used it as monotherapy [51, 75]. In a prospective, open-labeled, non-randomized trial including 10 HS patients to whom eight cycles of bortezomib were given; Gonzalez *et al.* found only a modest decline in HLA antibodies. Furthermore, two patients discontinued bortezomib secondary to its side-effects [76]. A randomized, placebo-controlled trial showed that bortezomib therapy did not show any significant benefit in GFR loss, DSA decline, and histologic changes despite substantial toxicity [77].

Carfilzomib is a second-generation proteosome inhibitor (PI), which is an IV epoxyketone. Its irreversible binding and non-boronated nature confer long-lasting inhibition of proteosome and reduced peripheral neurotoxicity compared to bortezomib [51, 74]. A small study including 13 HS patients who underwent desensitization based on carfilzomib in combination with PP showed an acceptable safety and toxicity profile while leading to significant bone marrow plasma cell depletion and anti‐HLA antibody reduction. However, after depletion, rebound was observed and antibody levels returned to baseline between days 81 and 141 [75]. Although promising as an agent for desensitization in KT, further studies preferably with additional agents preventing DSA rebound are needed to underpin the initial gains achieved with carfilzomib.

### Monoclonal anti-plasma cell antibodies

Daratumumab is a human immunoglobulin G1 mAb that targets CD38 transmembrane glycoprotein on the surface of plasma cells, natural killer (NK) cells, myeloid-derived suppressor cells, and B_reg_s [78, 79]. Alloantibody-producing plasma cells express CD38 at a higher level than other CD38^+^ hematopoietic cells [80]. Case reports and case series showed that daratumumab reduced DSA MFI strength, allowed for HLAi transplantation and improved severity of AMR [81, 82]. Nevertheless, antibody rebound, B_reg_ depletion, and induction of T-cell mediated rejection seems to be the current hurdles that limit its efficacy [83]. Pilon *et al.* conducted a single-center, open-label, phase 1/2 trial of daratumumab on adult patients who had been waiting for KT for at least 3 years and had a cPRA > 95% [84]. Although the authors found a profound decrease in cPRA, the number of HLA antibodies, and their MFI-max at 3 months after the first infusion, almost all parameters returned to baseline values within 6 months.

Isatuximab is a chimeric IgG1-kappa CD38 mAb [85]. In an open-label single-arm phase 1/2 study conducted at six centers in the USA and Spain, Vincenti *et al.* investigated safety and efficacy of isatuximab monotherapy in HS (cPRA > 80%) patients awaiting KT [86]. Twenty-three patients received isatuximab 10 mg/kg weekly for 4 weeks then every 2 weeks for 8 weeks. Isatuximab was well tolerated. It resulted in reduction of CD38^+^ plasmablasts, plasma cells, and NK cells and in a significant decline of HLA-specific IgG-producing mBCs. Most responders had decreases in HLA antibodies that were maintained for 26 weeks after the last dose. Although isatuximab demonstrated a durable decrease in some of the HLA antibodies, it had minimal effect on the overall cPRA values. Authors did not observe any T cell-mediated rejection in the transplanted patients (4 out of 23) treated with isatuximab as of study cut-off date.

CD38-targeting agents have been shown to reduce HLA antibodies [87], however, their efficacy is not consistent across all patients. Recently Torija *et al.* identified critical circulating mBC subset phenotypes (CD38 neg class-switch mBCs) that distinguish HS patients with successful serologic responses to CD38-targeting desensitization therapies from those who are low or non-responders, potentially guiding treatment decision-making [88].

### Co-stimulatory blockade combined with anti-plasma cell therapies

Vincenti *et al.* hypothesized that adding co-stimulation blockade to plasma cell depletion would thwart nodal B-cell and T-follicular helper (Tfh) expansion and provide sustained decline in circulating HLA antibodies [89]. Preliminary results of this ongoing ATTAIN study (NCT04827979) presented at the American Transplant Congress 2024, showed that a novel 10-week HLA desensitization regimen, involving daratumumab and belatacept, enabled DD-KT in three out of five waitlist candidates with a cPRA > 99.9%. Post-transplant DSA rebound has not been observed so far [90]. Jackson *et al.* conducted a pilot phase I/II trial study of carfilzomib with belatacept for desensitization in five HS (cPRA > 99%) patients. Early results of this study showed that the composite efficacy endpoints (elimination of one HLA antibody, ≥50% MFI reduction of ≥3 HLA antibodies, or KT with a previously incompatible donor) were achieved in two patients [91].

## IL-6 TARGETING DRUGS

IL-6 is an essential cytokine that modulates various inflammatory and immunomodulatory pathways [92, 93]. It takes part in induction of Tfh cells, germinal center creation, differentiation of naive B cells to plasma cells, and high affinity antibody production [94–96]. IL-6 also inhibits conversion of T-helper 17 cells to regulatory T cells, thereby promoting allograft injury and stimulates profibrotic cytokines [95]. These features of this pleiotropic cytokine draw attention from investigators to target it for desensitization in HS patients and to modulate acute and chronic AMR (cAMR) in KT recipients.

### Tocilizumab

Daligault *et al.* demonstrated that tocilizumab as a monotherapy significantly decreased the number and intensity of dominant HLA antibodies in HS kidney transplant candidates [97]. Nevertheless, only 1 out of 14 patients could be transplanted, indicating that tocilizumab as a monotherapy was not sufficient to proceed to KT. Jouve *et al.* examined the effect of tocilizumab as an add-on therapy to SOC desensitization (based on RTX and apheresis) in HS patients [98]. They found that tocilizumab as an add-on therapy (8 mg/kg monthly, six injections before SOC) may help control the post-transplant rebound of antibodies with elevated baseline MFIs. However, reductions in pre-transplant MFIs were not statistically significant compared to SOC alone.

### Clazakizumab

Clazakizumab is a humanized potent long acting IgG**_1_** antibody that binds and neutralizes human IL-6 [99]. In an open-label pilot study, Vo *et al*. enrolled 20 HS patients to determine the efficacy and safety of clazakizumab [100]. Pre-transplant, enrolled patients received desensitization with PP × 5/IVIG (2 g/kg × 1) and monthly clazakizumab 25 mg for 6 months starting at day 7. There were no significant reductions in HLA MFI values prior to treatment with clazakizumab. The post-transplant protocol included antibody induction with alemtuzumab, standard triple maintenance therapy (tacrolimus, mycophenolate mofetil and prednisone), and clazakizumab monthly for 6 months. Clazakizumab desensitization after PP + IVIg, provided significant reductions in HLA class I and II antibodies that allowed 18 of 20 patients to receive transplantation with no *de novo* DSA generation. Excluding the graft failure, the mean eGFR at 12 months for all 18 patients transplanted was 58 ± 29 ml/min/1.73 m^2^. IL-6 targeting drugs might have a potential future role in pre-transplant desensitization.

### Imlifidase

Imlifidase, the IgG-degrading enzyme purified from *Streptococcus pyogenes* (IdeS) is a recombinant cysteine protease that cleaves human IgG into its F(ab’)2 and Fc fragments inhibiting complement-dependent cytotoxicity and antibody-dependent cellular cytotoxicity [101]. Imlifidase impedes HLA antibody-mediated NK cell activation *in vitro* [102]. So far, imlifidase is the only therapy conditionally approved in Europe and Australia for desensitization of HS candidates for KT. Jordan *et al.* conducted a phase 1–2 open-label study which incorporated data from two studies in the USA (*n* = 14) and Sweden (*n* = 11) investigating the safety and efficacy of imlifidase in HS patients [101]. Patients in the USA received alemtuzumab for induction, while those in Sweden received horse anti-thymocyte globulin. Swedish patients, who did not receive IVIG and RTX following imlifidase, experienced DSA rebound beginning at 7 to 14 days after transplant. By contrast, patients in the US study, who all received IVІG and RTX following imlifidase, experienced less DSA rebound. Imlifidase reduced or eliminated DSAs and permitted HLA-incompatible KT in 24 of 25 patients. A follow-up, phase 2, multinational study further investigated the ability of imlifidase to convert a positive crossmatch test to negative among 19 HS (median cPRA 99.83%) KT recipients [103]. Crossmatch conversion was accomplished within 24 h in 17 of 18 patients who received a full dose of imlifidase. Post-dose, patients also received high-dose IVIG and RTX. DSAs most often rebounded 3–14 days post-imlifidase dose and 38.9% of patients had early biopsy-proven AMR. However, a 100% patient survival and 88.9% allograft survival were observed at 6 months post-transplant. Kjellman *et al.* published a report that summarizes clinical and immunologic outcomes of four single-arm, open-label phase 2 studies over a total of 39 crossmatch-positive KT recipients receiving imlifidase therapy as their desensitization protocol [104]. Three years after imlifidase-enabled desensitization and transplantation, the death-censored allograft survival was 84%, patient survival 90%, and mean eGFR was 55 ml/min/1.73 m^2^. Recently, same group reported a patient survival of 90% and death-censored graft survival of 82% after 5-years follow-up of the same patient population [105]. Very recently, Jaffe *et al.* reported a single-center case series showing that imlifidase-enabled HLAi-KTs have a good 5-year overall survival (87.5%) and death-censored graft survival (85.5%) rates with a favorable graft function [106]. Imlifidase was also tested in treatment of AMR. A randomized, open-label, multicenter, multinational trial conducted by Hallect *et al.* failed to demonstrate a meaningful clinical benefit of imlifidase in treatment of AMR compared to PP despite a faster and higher magnitude of DSA reduction [107]. In spite of an acceptable safety and efficiency profile, its high cost, development of neutralizing antibodies, and antibody rebound that necessitates other treatments (i.e. IVIG and RTX) are the current significant limitations of imlifidase use [108, 109].

## COMPLEMENT BLOCKADE

Experimental and clinical evidence and pathologic features suggest that one of the effector mechanisms of AMR is activation of the complement system by HLA antibodies inducing tissue damage and graft dysfunction [110–113].

### Eculizumab 

Eculizumab is a humanized mAb that targets terminal complement protein C5 and preventing the formation of the terminal complement complex. In a single-center pilot study, even though eculizumab did not have an impact on DSA levels early after transplantation, HS recipients of FC-XM positive LDKT who were given eculizumab suffered a much lower rate of early AMR (within first 3 months after transplant) compared to historical controls (7.7% vs 41.2% respectively) [38]. However, this lower incidence of early AMR in eculizumab-treated recipients did not result in improved long-term allograft survival [30, 114]. A phase 2, randomized, multicenter, open‐label study including sensitized recipients of LDKTs requiring desensitization did not find a significant difference in treatment failure rates between the eculizumab (9.8%) and the SOC (13.7%) groups. Likewise, no significant differences in patient or graft survival were observed between the two treatment groups [115]. A multicenter, international study comparing the current SOC (plasma exchange and IVIG) to terminal complement inhibition has demonstrated a significant decrease in the incidence of early AMR (within 3 months post-transplant) in patients with complement-activating anti-HLA DSAs but not in those with non-complement-activating DSAs [116]. Therefore, as an add-on to SOC desensitization, eculizumab may be used in the future to prevent post-transplant early AMR secondary to complement binding HLA antibodies in HS patients. However, eculizumab is an expensive drug with yet unclear long-term benefits; therefore, its role in desensitization protocols is uncertain.

### C1 esterase inhibitor (C1-INH)

C1 inhibitor (C1-INH) is a multifunctional member of the serpin family of protease inhibitors that take part in regulation of the classic complement and lectin pathways [117]. A randomized, placebo-controlled study conducted by Vo *et al.* evaluated C1-INH in HS renal transplant recipients for prevention of AMR [118]. Twenty HS patients desensitized with IVIG + RTX ± PP were enrolled and randomized 1:1 to receive plasma-derived human C1-INH or placebo intraoperatively. This RCT showed that C1-INH is safe and reduce C1q + HLA antibodies. Furthermore, no AMR episodes were detected during study period. A pilot phase 1 in-patient trial designed to assess the safety and tolerability profile of the anti-C1s mAb BIVV009 in KT recipients with late AMR showed that BIVV009 effectively blocked alloantibody-triggered classical pathway activation. However, short-course treatment had no effect on indices of activity in late AMR [119]. The available current evidence advocates the efficacy for complement inhibitors in treating AMR. However, to alleviate non-complement-associated but DSA induced pathways, combination therapies with complement inhibitors will presumably be needed.

## BAFF INHIBITORS

B cell-activating factor (BAFF) is a cytokine in the tumor necrosis factor family. BAFF enhances survival of immature and transitional B cells thereby taking part in B-cell maturation [120]. A pilot study to assess the use of belimumab (a humanized mAb that inhibits BAFF) as monotherapy to reduce preformed antibodies in sensitized candidates awaiting KT was terminated after enrolling eight patients because of lack of a global impact on the anti-HLA IgG alloantibodies [120, 121]. Abaticept is a mAb that targets both a proliferation inducing ligand (APRIL) and BAFF. In a sensitized murine model, abaticept use was associated with reduction of antibody secreting cells. However, this finding did not translate into decreased KT rejection rates [122]. The same group tried abaticept in a chronic rejection transplant mouse model. Despite the depletion of DSAs and mature B lymphocytes, abaticept failed to decrease KT rejection rates [123]. The authors attributed the significant cellular rejection observed in the abaticept treated animals to increased effector T lymphocyte and decreased regulatory T lymphocyte populations. APRIL/BAFF interact with three different receptors on plasma cells, which would oblige combination therapies: B-cell maturation antigen (BCMA), transmembrane activator and calcium-modulator and cyclophilin ligand interactor (TACI), and BAFF receptor (BAFF-R) [124].

## CAR T-CELL THERAPIES

### Chimeric antigen receptors

Autologous T cells, engineered to express chimeric antigen receptors (CARs) that target specific antigens has shown remarkable success in hematologic malignancies. Given the limited efficacy of the current desensitization methods and organ shortage, there are more attempts being made to try T cell-based therapies in the context of solid organ transplantation (SOT) [125]. Hill *et al.* found that CAR T-cell (CAR T) therapy given for hematologic malignancies could not reduce HLA antibodies, which are presumably sustained by CD19^−^ long-lived plasma cells [126]. Liu *et al.* constructed a B-cell maturation antigen-CD19 combination CAR (cCAR) that was found to be very effective in reducing DSA levels in patients with B-ALL, making them eligible candidates for stem cell transplant [127]. This first in-human phase 1 study of cCAR therapy suggested that sensitized candidates of SOT could also benefit from cCAR. Jarmi *et al.* demonstrated that MC10029 (anti-BAFF-R) CAR T cells derived from HS (cPRA > 98%) patients *in vitro* displayed cytotoxicity against their autologous B cells [128]. This finding suggests that CAR T-cell therapy is a viable option for desensitization in SOT [128]. An ongoing trial (NCT06056102) that is enrolling HS candidates (cPRA > 99.5%) for KT will evaluate the safety and efficacy of desensitization with chimeric antigen receptor T–B-cell maturation antigen (CAR T-BCMA) with CD19-targeted humanized CAR T cells (huCAR T-CD19). One remarkable advantage of CAR T-cell therapies over monoclonal antibodies is their longer duration of therapeutic effects [129]. However, these therapies are yet in their early phases of evolution with the current hurdles of development of cytokine release syndrome, lack of efficacy in IgG-producing long-lived plasma cells, and recognition by cytotoxic T lymphocytes capable of killing the infused cells [129].

### Chimeric HLA antibody receptor T cells

A new strategy introduced to target HLA-specific humoral immune response is the chimeric HLA antibody receptor (CHAR) T cells that eliminate HLA-directed B cells. Gille *et al.* developed CHAR constructs comprising the extracellular part of HLA-A2 or HLA-A3 coupled to CD28-CD3ζ domains. HLA-A2 and HLA-A3 CHAR T cells produced IFNγ and they were capable of specifically lysing hybridoma cells expressing HLA-A2- or HLA-A3-specific B-cell receptors [130]. Current desensitization strategies non-specifically target circulating antibodies and B cells. This study showed that CHAR T cells could provide precision immunotherapy to desensitize HS patients.

## NEONATAL FC RECEPTOR INTERFERENCE

An alternative method for desensitization is targeting the neonatal Fc receptor (FcRn), which is essential for maintenance of circulating IgG levels. Manook *et al.* tested the efficacy of targeting this IgG recycling mechanism using anti-FcRn mAb therapy in a sensitized non-human primate model [131]. This antibody reduced circulating DSAs, however, after initial decline, a rapid antibody rebound occurred. It is possible that the addition of proteosome inhibitors or IVIG to FcRn inhibition may overcome the limitations of anti-FcRn therapy in future trials.

## NOVEL COMBINATION THERAPIES

Even though considerable progress has been achieved in desensitization strategies, allograft survival of HS patients is still inferior than non-sensitized patients, an outcome mainly driven by persistence or *de novo* development of HLA antibodies post-transplant. In spite of significant advances, none of the desensitization strategies could have yet overcome the HLA barrier in HS patients with strong antibodies. Thus, it would be potentially germane to target multiple steps of DSA production in HS patients. Naive T cells are activated by the three signaling pathways including T-cell receptor interaction with the presented peptide (signal 1), co-stimulation (signal 2), and cytokine stimulation (signal 3) that induces clonal expansion [132]. AMR is regarded as the major driver of the immunologic graft loss in KT. Tfh cells and their interaction with B cells are needed for germinal center (GC) formation, and long-lasting antibody production [133–135]. Co-stimulation blockade targets the GC response since this interaction relies on many co-stimulatory signaling [136]. Kwun *et al.* postulated that CNI-based conventional maintenance regimen does not efficiently suppress Tfh cells during T-cell repopulation after transplant which results in a GC-driven antibody response [137]. Cytotoxic T lymphocyte associated protein 4 (CTLA4-Ig) inhibits Tfh cell differentiation by blocking CD28 co-stimulation, which is the mechanism proposed for its inhibition of *de novo* DSA responses to allograft [138]. However, CTLA4-Ig alone does not inhibit CD8^+^ effector/memory T-cell response, which may explain the high rate of acute cellular rejection seen in patients early after transplant who receive CTLA4-Ig without CNI [79, 139]. In a pre-clinical non-human primate model of allogeneic KT, Kubo *et al.* concluded that peri-transplant tocilizumab infusion attenuates co-stimulation blocker-resistant T cells in CTLA-Ig treated monkeys [140]. Guitares *et al.* constructed a human fusion recombinant protein with CTLA4 and programmed cell death 1-ligand L2 (PD-L2) domains called HYBRI, which can block the CD28-CD80 co-stimulatory pathway while stimulating the PD-1-PD-L2 co-inhibitory pathways. The authors showed that HYBRI protein could effectively prevent rejection and alleviate damage from warm ischemia reperfusion in rat models [141]. A phase 2 pilot study tested dual co-stimulation blockade of the CD28-CD80/86 and CD40-CD40L pathways to prevent allograft rejection in adults undergoing a low immunologic risk first KT [142].

## DESENSITIZATION IN DECEASED-DONOR KIDNEY TRANSPLANTATION

Another option to enhance the probability of KT is desensitization of sensitized candidates on the DD waiting list. Sensitized patients on the DD waiting list can be opted for pre-transplant desensitization when the DD kidney offer is likely within a rational time range (i.e. 1 year), which depends on the national scoring systems by which allocation of kidney grafts are formulated. Notwithstanding, desensitization of sensitized candidates on the wait list is expensive and laborious; in addition, entails HLA antibody surveillance.

### High-dose IVIG/RTX

Vo *et al.* found favorable KT rates in HS candidates desensitized with a high-dose IVIG + RTX protocol with reasonable post-transplant outcomes [39, 59] However, two other US studies did not confirm these results [143, 144]. High-dose IVIG/RTX (without PP) treatment may not be able to decrease the strength of alloantibodies and increase the transplant rates in patient groups with very high cPRA (>90%), high titers of DSA, and predominantly class II HLA antibodies.

### High-dose IVIG/RTX/PP

A French study including patients with preformed DSA and receiving DD kidneys showed that high-dose IVIG + RTX (IVIG 2 g/kg at days 0, 21, 42, and 63; RTX 375 mg/m^2^ at day 4) together with PP (was started immediately after transplant and continued three sessions weekly for 3 weeks thereafter) found acceptable short term graft outcomes (2/18 graft loss at a mean follow-up of 19 months) with a reasonable AMR rate (16.6%) [145]. PP/low-dose IVIG protocol is not suitable for DD-KT owing to the ambiguity about the timing of transplantation unless only one overnight treatment is performed pre-transplantation, the remaining being performed post-transplantation.

### Tocilizumab/IVIG

In a phase I/II single-center open-label pilot study, Vo *et al.* found that addition of the IL-6R antibody tocilizumab (8 mg/kg administered on day 15, then monthly for 6 months) to IVIG (on days 1 and 30) enabled transplantation with a reasonable safety profile in 5 of 10 sensitized (cPRA > 50%) patients who were previously failed desensitization with IVIG + RTX treatment [146].

## CONCLUSIONS

**Figure 3:**
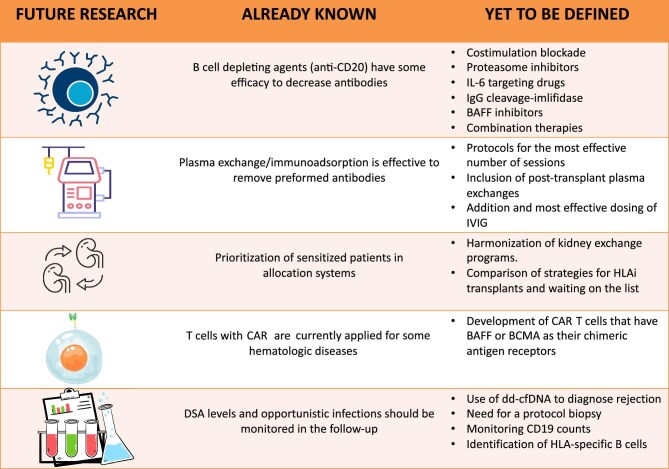
Desensitization in HLA-incompatible KT: current aspects, future trends, and research directions.

A well-designed KPD or kidney allocation system still may not accomplish access to transplantation for those very HS patients (cPRA > 99%). Currently, most of the crossmatch-positive KTs are successfully performed through desensitization, however, recurrent or *de novo* antibody-driven graft injury culminating in decreased allograft survival cannot be averted. Clinical trial designs including novel therapeutic agents capable of both reduction of antibodies and then prevention of their rebound are essential to augment the longevity of kidney transplants (Fig. 3). Recent trials of emerging therapeutics suggest that a rational combination of multiple agents acting on multiple stages of antibody formation and graft injury could have a promising future.

## Data Availability

Our paper has no associated data.
